# The prevalence and epidemiology of plasmid-mediated penicillin and tetracycline resistance among *Neisseria gonorrhoeae* isolates in Guangzhou, China, 2002–2012

**DOI:** 10.1186/s12879-015-1148-9

**Published:** 2015-10-09

**Authors:** Heping Zheng, Xingzhong Wu, Jinmei Huang, Xiaolin Qin, Yaohua Xue, Weiying Zeng, Yinyuan Lan, Jiangli Ou, Sanmei Tang, Mingheng Fang

**Affiliations:** Guangdong Provincial Centre for Skin Diseases and STIs Control and Prevention, Guangzhou, 510091 China

**Keywords:** *Neisseria gonorrhoeae*, Antibiotic, Susceptibility, Plasmid, Genotype

## Abstract

**Background:**

Gonococcal antimicrobial resistance is a global problem. Different resistance plasmids have emerged and spread among the isolates of *Neisseria gonorrhoeae* worldwide and in China. We conducted this study to monitor the plasmid-mediated penicillin and tetracycline resistance among *N. gonorrhoeae* isolates in Guangzhou from 2002 to 2012.

**Methods:**

Consecutive isolates of *N. gonorrhoeae* were collected from outpatients with gonorrhea attending the STD clinic in Guangdong Provincial Centre for Skin Diseases and STIs Control and Prevention. Penicillinase-producing *N. gonorrhoeae* (PPNG) isolates were analyzed by the paper acidometric method. Plasmid-mediated resistance to tetracycline in *N. gonorrhoeae* (TRNG) isolates was screened by the agar plate dilution method. Plasmid types were determined for TRNG and PPNG isolates using polymerase chain reaction (PCR). Minimum inhibitory concentrations (MICs) to penicillin and tetracycline were detected by the agar plate dilution.

**Results:**

Of 1378 consecutive *N. gonorrhoeae* isolates, 429 PPNG and 639 TRNG isolates were identified. The prevalence of PPNG, TRNG, and PPNG/TRNG increased from 18.3 to 47.1 % (*χ*^2^ = 31.57, *p* < 0.001), from 29.4 to 52.1 % (*χ*^2^ = 16.28, *p* < 0.001) and from 10.0 to 26.2 % (*χ*^2^ = 10.46, *p* < 0.001) between 2002 and 2012, respectively. Genotyping of plasmids among PPNGs showed that the majority (93.7 %) of the isolates were the Asian type plasmids, while the African type plasmid emerged in 2008 and rapidly increased to 14.0 % in 2012 (*χ*^2^ = 25.03, *p* < 0.001). For TRNGs, all 639 isolates carried the Dutch type plasmid. MICs of penicillin G and tetracycline persisted at high levels and the MIC_90s_ were 32-fold higher than the resistant cutoff point over 11 years. The prevalence rates of penicillin- and tetracycline-resistant *N. gonorrhoeae* varied from 90.9 to 91.1 % and from 88.3 to 89.3 % during 2002 to 2012, respectively.

**Conclusions:**

Resistance to penicillin and tetracycline among *N. gonorrhoeae* isolates remained at high levels in Guangzhou. The Asian type PPNG continued to spread and Dutch type TRNG was still the dominant strain. The African type PPNG has emerged and is spreading rapidly.

## Background

*Neisseria gonorrhoeae* is the causative agent of gonorrhea, which is the second most prevalent bacterial sexually transmitted infection globally. The emergence and spread of antibiotic resistant *N. gonorrhoeae* has posed a challenge for successful control of gonorrhea worldwide. In recent decades, resistance developed to the antimicrobials previously recommended for treatment of gonorrhea, including penicillin, tetracyclines and fluoroquinolones [1–3]. Treatment failures regarding extended-spectrum cephalosporins such as oral cefixime and injectable ceftriaxone have been documented in Japan and several European countries, as well as resistance to spectinomycin, leading to the concern of no effective antibiotics for gonorrhea therapy in the near future [4–10].

Two types of *N. gonorrhoeae* resistance to penicillin and tetracycline are identified. They are chromosomally mediated acquisition of mutated genes or loci and plasmid-mediated resistance. Plasmid-mediated resistance to penicillin is due to the production of penicillinase [11]. Three main resistance plasmids (R-plasmids) are associated with the worldwide emergence of penicillinase-producing *N. gonorrhoeae* (PPNG). These main R-plasmids include an “Asian” R-plasmid of 7.4 kb in length, and its deletion-derivatives “African” (5.6 kb) and “Toronto” (5.2 kb) [12]. Other types of penicillinase-producing plasmids have also been described in gonococci, e.g. Nimes, New Zealand, Australian and Johannesburg [12–15]. High level resistance to tetracycline (MIC > 16 mg/L) in gonococci are mediated by a *tet*M determinant carried on a 25.2 MDa conjugative plasmid [16]. The restriction endonuclease map of the 25.2 MDa conjugative plasmid from a tetracycline resistant *N. gonorrhoeae* (TRNG) strain imported from the United States has been found to differ from a map derived from a strain with the 24.5 MDa conjugative plasmid isolated in the Netherlands [17]. These two types of *tet*M carrying conjugative plasmids were designated as “American” and “Dutch” types, respectively. Subsequent epidemiological studies have suggested a worldwide distribution for TRNG isolates [18, 19]. Determination of plasmid-mediated penicillin and tetracycline resistance among *N. gonorrhoeae* is a useful epidemiologic tool for monitoring the movement or importation of isolates within a geographic region. However, the monitoring of these isolates in Asia is still limited.

Guangdong province in south China has over 100 million people, and one-quarter of them are migrant population. As a province that first opened to the world in 1980s, Guangdong experienced a rapid increase of STIs, including gonorrhea in the past three decades. In 2012, Guangdong reported 18,004 gonorrhea cases to the national disease reporting system accounting for 18.9 % of the total cases identified in China [20]. Even though we have developed a gonorrhea reporting system, we still lack a comprehensive system that systematically monitors the resistance of *N. gonorrhoeae*. Our early survey showed that the prevalence of penicillin resistance had increased from 57.2 to 81.8 % and PPNG had rapidly increased from 2 to 21 % during 1996–2001 [21]. In order to monitor the change of penicillin- and tetracycline-resistant *N. gonorrhoeae*, and the change of PPNG and TRNG type, we conducted this study in Guangzhou, the capital city of Guangdong, from 2002 to 2012.

## Methods

### Ethics statement

The study has been approved by ethics committee of Guangdong Provincial Centre for Skin Diseases and STIs Control and Prevention. All patient data were anonymously reported, with no possibility of connecting the isolates to individual patients. In addition, the specimens used in this study were all clinical residual specimens, and no personal information was collected, so we did not need to ask for the participants to sign an informed consent. We have no longer have any contact with them.

### Bacterial isolates

This study was conducted at Guangdong Provincial Center for Skin Disease and STIs Control and prevention. One thousand three hundred seventy eight consecutive isolates of *N. gonorrhoeae* were collected from patients diagnosed with gonorrhoea attending our STD clinic from 2000 to 2012. The isolates were cultured on Thayer-Martin agar and identified as *N. gonorrhoeae* based on colony morphology, Gram staining, oxidase test and carbohydrate degradation tests (all sugars were supplied by FLUKA company) as recommended by WHO [22]. The strains were preserved in freeze-dried skimmed milk and stored at −70 °C until use. Antimicrobial susceptibility and plasmid-mediated resistance were analyzed in December of each year. All the isolates were revived successfully.

The WHO *N. gonorrhoeae* reference strains A, E, G and WHO-97QA3 were kindly provided by Dr Yin Yueping (National Center for STD control, China). PPNG control plasmids, pJD4 (Asian type), pJD5 (African type), and pJD7 (Toronto type) were kindly provided by Dr. J R Dillon (University of Saskatchewan, Canada). In addition, we used WHO-G with American type plasmid and WHO-97QA3 with Dutch type plasmid as the TRNG control plasmids.

### MIC detection and Interpretation of susceptibility

All isolates were examined for susceptibility to penicillin G (SmithKline Beecham Limited) and tetracycline (Sigma-Aldrich Co. LLC.) by agar plate dilution method for MIC and antimicrobial susceptibilities were interpreted according to the criteria developed by WHO WPR Resistance Surveillance Programme guidelines [22]. For penicillin, isolates with MICs ≥ 1.0 mg/L were classified as resistant, isolates with MICs of 0.6–0.50 mg/L as intermediate sensitive, and MICs ≤ 0.6 mg/L as sensitive. Isolates with MICs ≤ 0.5 mg/L to tetracycline were classified as sensitive and isolates with MICs > 1 mg/L as resistant. WHO strains were used as quality controls; the MIC of penicillin to WHO A, E (PPNG) and G were 0.008 mg/L, MIC > 2 mg/L and 0.5 mg/L, respectively and the MIC of tetracycline to WHO A, E and G (TRNG) were 0.25 mg/L,1 mg/L and 32 mg/L, respectively.

### Detection of PPNG and TRNG

PPNG isolates were analyzed by the paper acidometric method [22]. WHO reference strains A and E were used as negative and positive controls, respectively. TRNG isolates were screened by the agar plate dilution method with the criteria of MIC ≥ 16 mg/L [23].

### Genotyping of PPNG and TRNG

DNA extraction [21]: One hundred microliters of the *N. gonorrhoeae* isolates suspension (equivalent to McFarland No. 1 standard) in distilled water after 18 h’ incubation were centrifuged at 14,000 g for 30 min. The pellet was resuspended in 50 μL of lysis buffer (10 mmol/L Tris HCl pH 7.5, 1 mmol/L EDTA pH 8.0, 0.1 % Triton and 3000 U/L of proteinase K) and then incubated at 100 °C for 15 min. The supernatant were then used as a template for PCR amplification.

The Asian (7426 bp), African (5599 bp) and Toronto (5154 bp) type plasmids of PPNGs were differentiated by the PCR assays [24]. Four pairs of PCR primers were used for TEM-1 gene. The *tet*M gene of TRNG was amplified to identify American (AR) and Dutch (DR) variant [25]. A universal forward primer (UF) that hybridizes with both variants was combined with reverse primers specific to each variant (AR and DR). The primer sequences were derived from the sequences of pOZ100 (UF, AR) and pOZ101 (DR). All primers described in Table 1 were synthesized by Invitrogen Bio. Co. (Shanghai, China).

**Table 1:** Primers used for the detection of TEM-1 and *tet*M in *N. gonorrhoeae*

Gene	Primer	Direction	Primer sequencea (5′-3′)	Reference
TEM-1	BL1	Forward	^1545^TACTCAATCGGTAATTGGCT^1564^	Palmer HM et al.^24,a^
	BL2	Forward	^3606^CACCTATAAATCTCGCAAGC^3625^	
	BL3	Reverse	^4564^CCATAGTGTTGAGTATTGCGAA^4543^	
	BL4	Reverse	^6528^TCATTCGTGCGTTCTAGGA^6510^	
*tet*M	UF	Forward	^825^CTCGAACAAGAGGAAAGC^842^	Turner A et al.^25^
	AR	Reverse	^1602^GCATTCCACTTCCCAAC^1586^	
	DR	Reverse	^1267^TGCAGCAGAGGGAGG^1253^	

^a^Positions are based on the Asian plasmid, Genbank accession number U20374

Four microliters of each extracted DNA from either reference or clinical isolates were used for amplification in a 50 μL solution containing: 200 μmol/L (each) dATP, dCTP, dGTP and dTTP; 50 μmol/L KCl; 10 mM Tris HCl (pH 8.4); 1.5 μmol/L MgCl_2_; 0.5 μmol/L of each oligonucleotide primer and 1.25 U of Taq DNA polymerase (Takara, Japan). Thirty-five cycles of amplification were performed in a thermocycler (Perkin-Elmer 9600). Each cycle consisted of 30 s of denaturation at 95 °C, 30 s of annealing at 55 °C and 1 min of extension at 72 °C. PCR products were analyzed by electrophoresis in 1 % *w*/*v* agarose and visualized by ultraviolet fluorescence after ethidium bromide staining.

### Statistical analysis

In this study, SPSS 18.0 (IBM) was used for all statistical analyses. Descriptive analyses were conducted to determine the distribution of genotype and resistance. In addition, we also performed trend analysis to test the trend of resistance during the study period.

## Results

### PPNG and TRNG prevalence

Of the 1378 consecutive gonococci isolated from 2002 to 2012, 429 PPNG isolates were identified by paper acidimetric method and 639 TRNG isolates were identified by agar plate dilution method. The prevalence of PPNG, TRNG, and PPNG/TRNG increased from 18.3 to 47.1 % (*χ*^2^ = 31.570, *p* < 0.001), from 29.4 to 52.1 % (*χ*^2^ = 16.282, *p* < 0.001) and from 10.0 to 26.2 % (*χ*^2^ = 10.462, *p* < 0.01) during the study period, respectively (Table 2).

**Table 2:** The distribution of types of TEM-1 and *tet*M among PPNG and TRNG isolates 2002–2012

Year	No.of isolates	PPNG (%)				TRNG (%)			PPNG/TRNG (%)
		No. of isolates	Type of TEM-1			No. of isolates	Type of *tet*M		
			Asain	African	Toronto		Dutch	American	
2002	180	33 (18.3)	33 (100)	0	0	53 (29.4)	53 (100)	0	18 (10.0)
2003	115	31 (27.0)	31 (100)	0	0	50 (43.5)	50 (100)	0	20 (17.4)
2004	90	21 (23.3)	21 (100)	0	0	44 (48.9)	44 (100)	0	8 (8.9)
2005	76	18 (23.7)	18 (100)	0	0	35 (46.1)	35 (100)	0	14 (20)
2006	104	27 (26.0)	27 (100)	0	0	40 (38.5)	40 (100)	0	19 (20.2)
2007	95	30 (31.5)	30 (100)	0	0	41 (43.6)	41 (100)	0	29 (23.6)
2008	183	75 (42.0)	74 (99.7)	1 (1.3)	0	108 (59.0)	108 (100)	0	48 (26.2)
2009	151	45 (29.8)	43 (96.6)	2 (4.4)	0	85 (56.3)	85 (100)	0	25 (16.7)
2010	136	51 (37.5)	44 (87.3)	7 (13.7)	0	67 (49.3)	67 (100)	0	33 (24.3)
2011	127	41 (32.3)	33 (81.5)	8 (19.5)	0	53 (41.7)	53 (100)	0	15 (11.8)
2012	121	57 (47.1)	48 (84.2)	8 (14.0)	1 (1.8)	63 (52.1)	63 (100)	0	32 (26.2)
Total	1378	429 (31.1)	402 (93.7)	18 (6.1)	1 (0.2)	639 (46.4)	639 (100)	0	261 (18.9)

### TEM-1 and *tet*M gene genotyping

The identified PPNGs and TRNGs were further genotyped according to the lengths of TEM-1 and *tet*M PCR production (Table 2). The results showed that Asian type plasmids dominated the *N. gonorrhoeae* isolates during the study period. However, African type plasmids emerged in 2008 (1.3 %) and quickly increased to 14.0 % in 2012 (*χ*^2^ = 25.029, *p* < 0.001). In addition, one Toronto type was identified in 2012. All of 639 TRNGs carried the Dutch type plasmids.

### MICs of penicillin G and tetracycline

The distribution of MIC_50_ and MIC_90_ of penicillin G and tetracycline from the 1378 consecutive isolates are summarized in Table 3. MICs of penicillin G and tetracycline persisted at high level and the MIC_90s_ were 32-fold higher than the resistant cutoff point over 11 years. The penicillin-resistant prevalence was maintained at high levels (90.9 % in 2002 to 91.1 % in 2012) as were for tetracycline resistance (88.3 % in 2002 to 89.3 % in 2012).

**Table 3:** Susceptibilities of *N. gonorrhoeae* to Penicillin G and Tetracycline 2002–2012 (mg/L)

Year	No.of isolates	Penicillin G^a^						Tetracycline				
		MIC_50_	MIC_90_	MIC range	S (%)	I (%)	R (%)	MIC_50_	MIC_90_	MIC range	S (%)	R (%)
2002	180	4	>32	0.03– >32	1 (0.6)	15 (8.3)	164 (91.1)	2	>16	0.125– >16	21 (11.7)	159 (88.3)
2003	115	16	>32	0.06– >32	0 (0.0)	3 (2.6)	112 (97.4)	2	>16	0.5– >16	23 (20.0)	92 (80.0)
2004	90	4	>32	0.125– >32	0 (0.0)	8 (8.9)	82 (91.1)	4	>16	0.5– >16	12 (13.3)	78 (86.7)
2005	76	16	>32	0.25– >32	0 (0.0)	5 (6.6)	71 (93.4)	4	64	<0.5– >64	15 (19.7)	64 (84.3)
2006	104	8	>32	>32–0.125	0 (0.0)	5 (4.8)	99 (95.2)	8	16	0.5– >64	20 (19.2)	84 (80.8)
2007	95	8	>32	0.06– >32	4 (4.2)	9 (9.5)	82 (86.3)	32	>32	<0.5– >32	17 (17.9)	78 (82.1)
2008	183	8	>32	<0.06– >32	2 (1.1)	16 (8.7)	165 (90.2)	16	>32	<0.25– >32	12 (6.6)	171 (93.4)
2009	151	1	>32	0.125– >32	1 (0.7)	44 (29.1)	106 (70.1)	32	>32	0.25– >32	13 (8.7)	138 (91.4)
2010	136	2	>32	0.125– >32	1 (0.7)	55 (40.4)	80 (58.9)	8	>32	<0.25– >32	17 (12.5)	119 (87.5)
2011	127	2	>32	<0.03– >32	1 (0.8)	22 (17.3)	104 (81.9)	4	>32	<0.125– >32	0 (0.0)	127 (100)
2012	121	4	>32	0.25– >32	0	11 (9.1)	110 (90.9)	4	>32	0.125– >32	13 (10.7)	108 (89.3)

^a^MIC mg/L, *S* sensitivity, *I* intermediate sensitivity, *R* resistance

## Discussion

Globally, the increasing proportion of PPNG and TRNG has become a serious concern. PPNG was initially found in clinical isolates in Thailand and in the United Kingdom in 1976 [11, 26], and TRNG was firstly found in the United States in 1985 [27]. Since then, more PPNG and TRNG were identified and reported worldwide. Epidemiological studies showed that countries in East Asia have significantly different distributions of PPNG, for example, 55 % in India, 33 % in Pakistan, 86 % in Bhutan in 2007–11 [28], and 79.3 % in Thailand in 2005–7 [29]. In Japan, among the 719 *N. gonorrhoeae* isolates isolated from January 2000 to December 2008, only 10 PPNG isolates (1.4 %) were found [30]. In Nanjing, China, PPNG and TRNG were reported to increase from 8.0 to 44.4 % and from 1.8 to 32.8 % respectively during 1999–2006 [31]. A high PPNG prevalence of 51.3 and 49.2 % were maintained while a TRNG increased from 19.6 to 42.1 % was observed in the neighboring city of Shanghai over two time periods, 2004–2005 and 2008–2011 [32]. In Guangzhou, the prevalence of PPNG increased yearly from 2 to 21.8 % and TRNG from 1.5 to 27.2 % during 1996–2001 [21]. In the present study, continuing increases were found from 18.3 to 47.1 % for PPNG and from 29.4 to 52.1 % for TRNG in 2002–2012.

In this study, we detected the Asian, African, and Toronto plasmids among our clinical isolates. The majority (93.7 %) of the consecutive PPNGs were genotyped as the Asian plasmid, which was similar to the results in Nanjing, China, in 1999–2006 [31]. However, the African type plasmid which was found in 2008 rapidly increased to 19.5 % in 2011 and 14.0 % in 2012; one Toronto type was first isolated in 2012. In contrast, different distributions of R-plasmid types were reported in Bangkok, Thailand, with 82.3 % African, 8.3 % Asian and 9.3 % Toronto types in 2005–7 [29], 100 % African type in Bangladesh in 2012 [33], 50 % African, 40 % Asian and 10 % Toronto types in Japan during 2000–8 [30] and 35.2 % African and 44.4 % Toronto types in South Africa in 2008 [34]. Our results showed a different trend of R-plasmid types in Guangzhou from 2002 to 2012. It is possible that the change over time is the result of more and more people from Africa and eastern Asian migrating and doing commercial business in Guangzhou.

High-level tetracycline resistance in *N. gonorrhoeae* is mediated by the *tet*M determinant. During 1988 to 1995, 518 isolates of TRNG isolated in the United Kingdom but originating from infections acquired in 39 countries worldwide were collected and typed by the *tet*M PCR assay. The results showed various distribution of *tet*M types, 98.3 % (59/60) American and 0.7 % (1/60) Dutch type in Africa, 40 % (4/10) American and 60 % (6/10) Dutch type in North, Central and South America, 64.3 % (36/56) American and 35.7 % (20/56) Dutch type in the Caribbean, 82 % (248/306) American and 18 % (58/306) Dutch type in Europe, and 100 % (35/35) Dutch type in the Far East [25]. In addition, Canada also reported that Dutch type *tet*M gene predominated (79.35 %) from 1986 to 1997 [35]. In recent years, 24 % (37/154) Dutch and 76 % (117/154) American type were found in South Africa in 2008 [34]. In Bangladesh,98.7 % (377/382) Dutch and 1.3 % (5/382) American type of TRNG were reported in 2012 [33]. However, in our study, we only found the Dutch type TRNG in the past 11 years. Similar results were reported in Nanjing in 2001–6 [31], Shanghai in 2004–5 [36] and Chengdu in 1999–2002 [37]. This finding further confirmed that Dutch type TRNG was dominant in China.

Penicillin and tetracycline have not been used as the first- or second-line therapeutic agents to treat gonorrhea for many years. During 2002–2012, penicillin and tetracycline had maintained high MICs in Guangzhou; this further supports the decision not to use penicillin and tetracycline to treat gonorrhoea. MIC_90s_ of penicillin G and tetracycline already reached at least 32-fold of the resistant cutoff point and were largely in accordance with previous surveys conducted in Southeast Asia. The rates of resistance to penicillin and tetracycline were much higher than the findings of studies conducted in Pakistan (86.8 % penicillin resistance and 77.6 % tetracycline resistance in 2009) [38], India (68 % penicillin and 55.4 % tetracycline resistance) and Vietnam (48 % penicillin and 82 % tetracycline resistance) in 2011 [39]. These rates indicated that South China might have even bigger challenges regarding resistant *N. gonorrhoeae*. In view of increasing resistance to cephalosporins such a cefixime and ceftriaxone [40], it is recommended that all cases of gonorrhea should also be covered for concomitant chlamydial infection as dual antibiotics might delay the onset of cephalosporin resistance [41]. Macrolide resistances *N. gonorrhoeae* is relatively low in many countries such as United States (0.04 %) [42]: and United Kingdom (1.6 % in England and Wales) [43] although cases have been reported [42, 44, 45]. In such cases, the choice for covering chlamydial infection should be a macrolide such as azithromycin rather than tetracycline such as doxycycline because of high rates of tetracycline resistance worldwide as well as in China. Furthermore, there is in vitro evidence of synergy between azithromycin and cephalosporin as well as more effective eradication for pharyngeal gonorrhea [46].

## Conclusions

In summary, the levels of resistance to penicillin and tetracycline were consistently high in Guangzhou. PPNG and TRNG have rapidly increased, in which, the Asian type PPNG has continued to further spread and the Dutch type TRNG is still the dominant strain in Guangzhou. In addition, the African type PPNG has increased in recent years, while Toronto type had emerged in 2012.

## Abbreviations

*N. gonorrhoeae*: *Neisseria gonorrhoeae*
MICs: Minimum inhibitory concentrations
PPNG: Penicillinase producing *N. gonorrhoeae*
TRNG: Tetracycline resistant *N. gonorrhoeae*
STD: Sexually Transmitted Disease
STIs: Sexually Transmitted Infections
